# Species-dependent differences of embryonic stem cell-derived neural stem cells after Interferon gamma treatment

**DOI:** 10.3389/fncel.2012.00052

**Published:** 2012-11-08

**Authors:** Janine Walter, Marcel Dihné

**Affiliations:** ^1^Department of Neurology, Heinrich-Heine-University DüsseldorfDüsseldorf, Germany; ^2^Department of Neurology and Epileptology, Hertie Institute for Clinical Brain Research, Eberhard-Karls-UniversityTübingen, Germany

**Keywords:** human neural stem cells, inflammation, interferon-gamma, neurodegeneration, IFNγ

## Abstract

Pluripotent stem cell (pSC)-derived, neural stem cells (NSCs) are actually extensively explored in the field of neuroregeneration and to clarify disease mechanisms or model neurological diseases *in vitro*. Regarding the latter, proliferation and differentiation of pSC-derived NSCs are investigated under the influence of a variety of different substances among them key players of inflammation. However, results generated on a murine genetic background are not always representative for the human situation which increasingly leads to the application of human cell culture systems derived from human pSCs. We investigated here, if the recently described interferon gamma (IFNγ)-induced dysregulated neural phenotype characterized by simultaneous expression of glial and neuronal markers on murine NSCs (Walter et al., 2011, 2012) can also be found on a human genetic background. For this purpose, we performed experiments with human embryonic stem cell-derived NSCs. We could show that the IFNγ-induced dysregulated neural phenotype cannot be induced in human NSCs. This difference occurs, although typical genes like signal transducers and activators of transcription 1 (Stat 1) or interferon regulatory factor 9 (IRF-9) are similarly regulated by IFNγ in both, murine and human populations. These results illustrate that fundamental differences between murine and human neural populations exist *in vitro*, independent of anatomical system-related properties.

## Introduction

Neural stem cells (NSCs) are multipotent and can give rise to the three principle cell types found in the adult mammalian brain, namely neurons, astrocytes, and oligodendrocytes. *In vivo*, they can be found in the hippocampus and subventricular zone where they mainly give rise to new neurons in the dentate gyrus and olfactory bulb, a process called neurogenesis (Kempermann, 2002, 2011). *In vitro*, they can be kept under proliferative conditions exhibiting immature neural markers like nestin and Sox2 due to the stimulus of growth factors, or differentiate to the above mentioned mature cell types when growth factors are withdrawn (Temple and Qian, 1996; Temple, 2001). Various regenerative approaches are aimed at using the capacity of NSCs as a therapeutic agent by transplanting them into injured brain regions (Barker et al., 2003; Harrower and Barker, 2005; Gogel et al., 2011). Additionally, NSCs from various sources are increasingly used to simulate diseases *in vitro* in order to set up model systems that can easily be manipulated and investigated. A specific and important advantage of *in vitro* model systems for neurological diseases is the possibility to use a human genetic background on the basis of human pluripotent stem cells (pSCs) in order to exclude, for instance, murine-specific phenomena. One interesting question, that could be clarified *in vitro* under controlled conditions, is how inflammatory processes that consistently occur in various neurological diseases act on NSCs. As *in vivo* neurogenesis is more and more accepted to contribute to processes like, for instance, memory function (Deng et al., 2009, 2010), it appears to be of interest to characterize effects of pathological processes such as inflammation on NSCs. Inflammation itself may lead to damage in brain tissue and represents either a primary disease entity or a secondary phenomenon following (Campbell, 2005), for instance, cerebral ischemia (Whitney et al., 2009). Interferon gamma (IFNγ), a pro-inflammatory key player, is a cytokine that is secreted by various cell types such as cytotoxic CD8^+^ T-cells, natural killer cells (Griffin, 1997), astrocytes, fibroblasts, and endothelial cells (Rady et al., 1995; De Simone et al., 1998; Wei et al., 2000). IFNγ signaling takes place via the IFNγ receptor which consists of two chains, situated in the cell membrane with an extra- and intracellular part (Schreiber and Farrar, 1993). The structure and the amino acid sequence of the murine and the human IFNγ protein and its receptor differ, although the physiological function remains the same (Farrar and Schreiber, 1993). These structural differences are leading to species-specific IFNγ—IFNγ receptor interactions with human IFNγ affecting only human and other primate cell types and vice versa (Schreiber et al., 1992; Schroder et al., 2004). IFNγ receptors were found on murine NSCs and therefore, effects of IFNγ on murine NSCs and related alterations in neurogenesis *in vivo* (Kim et al., 2002; Lin et al., 2004; Sweeten et al., 2004; Wang et al., 2004, 2008) and *in vitro* (Kim et al., 2007; Makela et al., 2010) were excessively explored. However, only little is known about the response of human NSCs (hNSCs) to IFNγ. We previously investigated effects of IFNγ on murine embryonic day 14-derived stem/precursor cells (msNSPCs) and murine embryonic stem cell-derived NSCs *in vitro* (Walter et al., 2011, 2012) (both populations are referred to as mNSCs in the following text). Predominantly in proliferative mNSC cultures, we found that IFNγ leads to a dysregulated phenotype, characterized by synchronous expression of neuronal and glial markers despite the presence of growth factors. This was accompanied by an unusual electrophysiological phenotype on single cell level preventing the ability to form synchronously bursting functional neuronal networks after differentiation. We also demonstrated an IFNγ-related significant up-regulation of sonic hedgehog (SHH) and Stat 1, key down-stream signals that are important for induction of the above mentioned phenotype (Walter et al., 2012). To assess the relevance of these findings with respect to the human situation, we (1) treated human embryonic stem cell-derived hNSCs with this pro-inflammatory cytokine and (2) measured IFNγ concentrations in cerebrospinal fluid (CSF) specimens collected from patients suffering from different nervous system diseases.

## Results

### hNSCs express IFNγ receptor I and II

**Figure 1:**
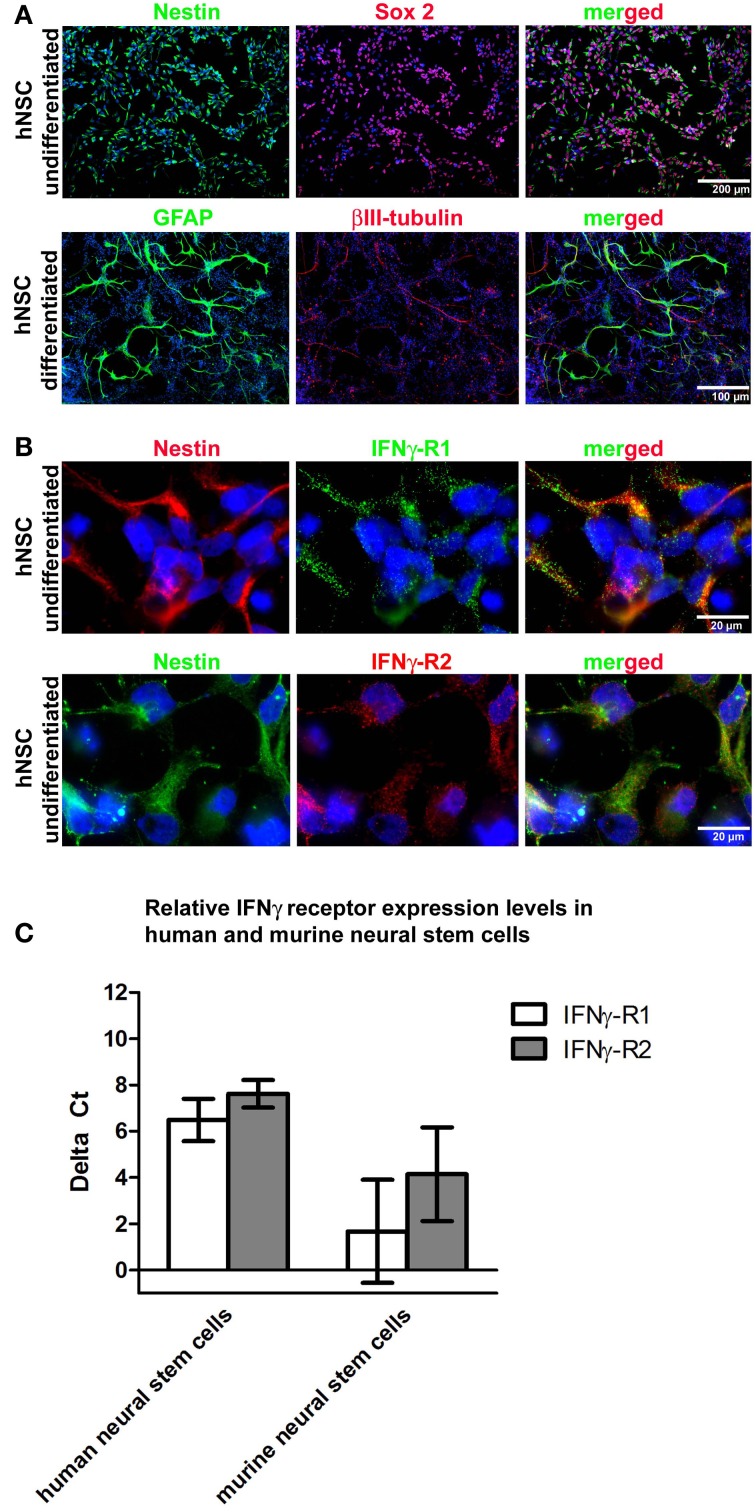
**hNSC express IFNγ-RI and IFNγ-RII.** In **(A)** representative photomicrographs of undifferentiated and differentiated hNSCs (Wicell h9 line) are given. To characterize the undifferentiated stage, proliferating cells under the influence of growth factors were seeded and then immediately PFA fixed for immunocytochemistry. To characterize the differentiated stage, cells were seeded and differentiated for four weeks with an initial stepwise reduction of growth factors. In the upper panel immunocytochemical stainings against the neural precursor markers nestin and Sox2 in undifferentiated hNSCs are given demonstrating that the pre-differentiation protocol which starts with pluripotent stem cells yields populations highly enriched in neural precursor cells. In the lower panel immunocytochemical stainings against βIII-tubulin and GFAP in differentiated hNSCs are given demonstrating the terminal differentiation of hNSCs into neurons and astrocytes. In **(B)** representative photomicrographs of undifferentiated hNSCs (Wicell h9 line) are given. A co-immunocytochemical staining against nestin and IFNγ-RI is given in the upper panel and against nestin and IFNγ-RII in the lower panel showing that both IFNγ receptor subunits are expressed in undifferentiated hNSCs. In **(C)** gene expression levels of IFNγ-RI and IFNγ-RII in undifferentiated hNSCs (Wicell h9 line) and mNSCs is shown.

In a first step, we immunocytochemically characterized the hNSC population generated from immature pluripotent embryonic stem cells. After neural pre-differentiation, almost all cells expressed nestin and most cells (>80%) expressed both, nestin and Sox2 (Figure 1A). After withdrawal of bFGF, NSCs terminally differentiated into βIII-tubulin^+^ neurons or GFAP^+^ astrocytes (Figure 1A). As the expression of membrane-bound IFNγ receptors (2 IFNγ receptor-1 chains and 2 IFNγ receptor-2 chains) is necessary to transduce the IFNγ signal, we performed immunocytochemical labelings against both receptor chains and were indeed able to demonstrate their expression (Figure 1B). We further investigated the expression levels of both receptor chains on mRNA level by means of real-time quantitative PCR. We compared these data with those generated on a murine background and found, that murine and human NSCs did not show significant differences (Figure 1C). Data were generated with the Wicell H9 line. We also confirmed these findings by using the HUES 6 line (data not shown).

### hNSCs do not express the dysregulated phenotype after IFNγ exposure

**Figure 2:**
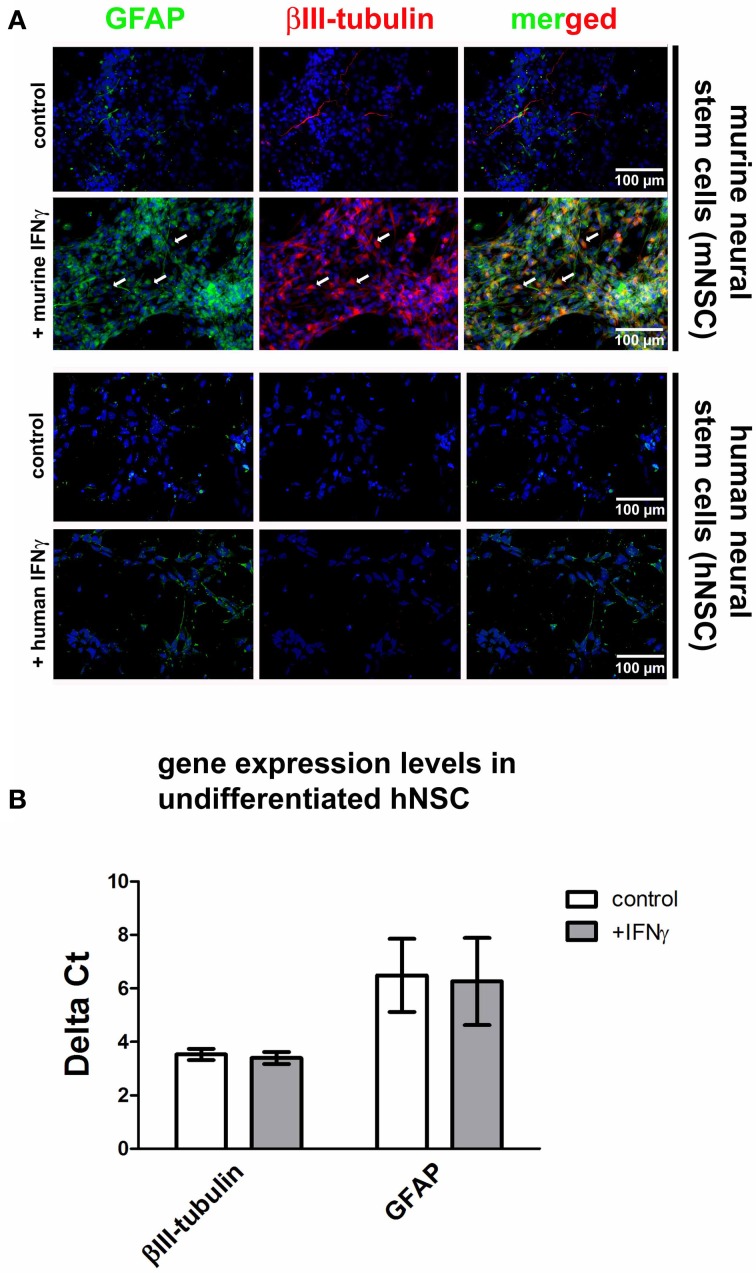
**Effects of IFNγ on hNSCs and mNSCs.** In **(A)** photomicrographs of undifferentiated hNSCs (Wicell h9 line) and mNSCs are given. A βIII-tubulin and GFAP co-staining under control conditions and after IFNγ treatment is shown. The upper to panels show mNSCs, the lower two panels show hNSCs. In mNSCs, IFNγ typically leads to a strong up-regulation of neuronal (βIII-tubulin) and glial (GFAP) markers with many cells simultaneously expressing both markers (arrows and the merged picture). In hNSCs no IFNγ-induced up-regulation of these markers was detected. In **(B)** gene expression levels of βIII-tubulin, GFAP, and SHH in undifferentiated hNSCs (Wicell h9 line) either under control conditions or after IFNγ treatment are shown. The results demonstrate that there is, also on mRNA level, no IFNγ-induced up-regulation of cell type-specific markers.

One major characteristic of the IFNγ-induced mNSC dysregulation is the coexpression of neuronal and glial markers under the influence of growth factors that normally hold NSCs in an immature and proliferating state (Figure 2A). This phenomenon is visible after a 3-days treatment with 1000 U/ml IFNγ and leads to a portion of around 39% of all cells that co-express GFAP and βIII-tubulin. The detailed characterization of this phenomenon is published elsewhere (Walter et al., 2011). However, when immature and proliferating hNSC populations under the influence of growth factors were treated with human recombinant IFNγ in identical concentrations compared to the murine situation, we were not able to detect this phenomenon (Figure 2A). These results were also confirmed on mRNA level (Figure 2B). Data were generated with the Wicell H9 line. We also confirmed these findings by using the HUES 6 line (data not shown).

### IFNγ-regulated genes in hNSCs and effects of IFNγ on the population size of hNSCs

**Figure 3:**
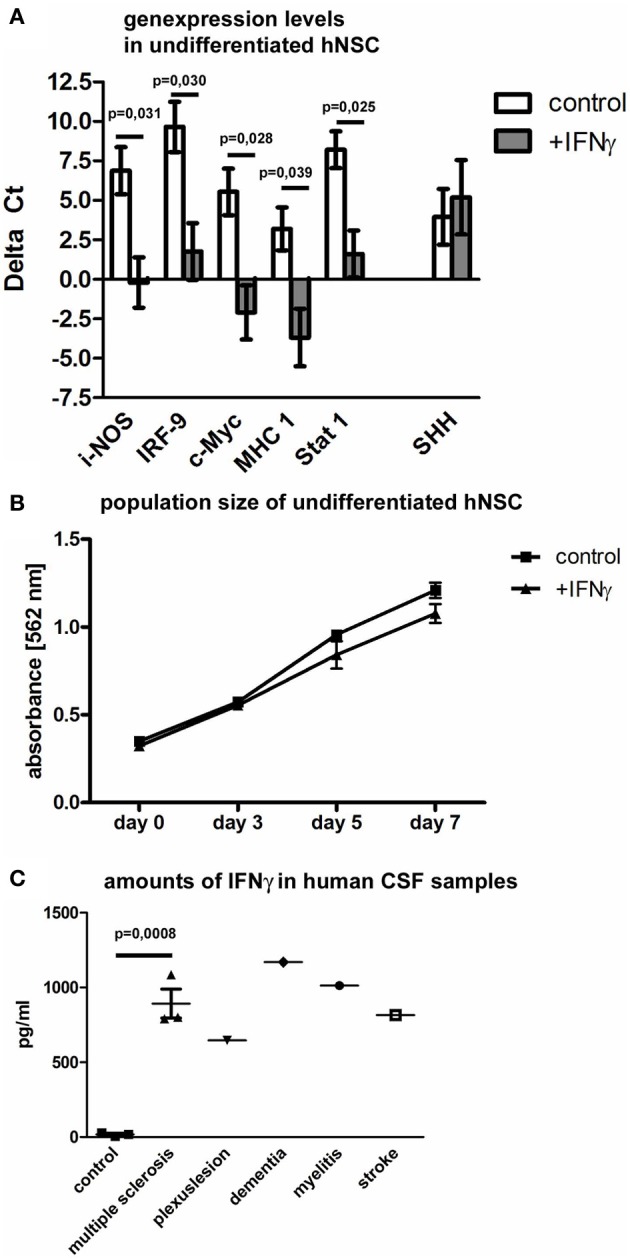
**Down-stream gene regulation and population size of hNSCs after IFNγ exposure and *in vivo* levels of IFNγ in patients CSF samples.** In **(A)** gene expression levels in hNSCs (Wicell h9 line) of i-NOS (inducible nitric oxid synthase), IRF-9 (interferon regulatory factor 9), c-Myc, MHC1 (major histocompatibility complex 1), and Stat 1 (signal transducers and activators of transcription 1) are given either under control conditions or after IFNγ treatment. Results show that IFNγ significantly up-regulates important down-stream factors except of SHH. Values are means ± standard error of mean (SEM). Experiments were performed in triplicate and repeated independently at least three times. In **(B)** population size of hNSCs (Wicell h9 line) over 7 days is measured by means of an MTT-assay. Population sizes of two groups, hNSCs under control conditions or after IFNγ treatment, are compared. Results show that IFNγ does not affect the population size of hNSCs. Values are means ± standard error of mean (SEM). Experiments were performed in triplicate and repeated independently at least three times. In **(C)** amounts of IFNγ in clinical CSF samples are measured by ELISA in comparison to CSF samples of control patients. For statistical testing of the multiple sclerosis group, 3 control patients were compared with 3 multiple sclerosis patients [unpaired two-tailed *t*-test, value is mean ± standard error of mean (SEM)].

As previously reported, the genes for inducible nitric oxid synthase (i-NOS) (Komatsu et al., 1996), IRF-9 (Ousman et al., 2005), c-Myc (Ramana et al., 2000), major histocompatibility complex 1 (MHC 1) (Johansson et al., 2008) and Stat 1 (Lehtonen et al., 1997) are significantly regulated in murine cells after IFNγ exposure. We now investigated the expression of these down-stream signaling genes after IFNγ exposure in hNSCs by means of real-time quantitative PCR. We found all of these genes to be significantly up-regulated after IFNγ exposure also in hNSCs (Figure 3A). As previously reported, we also found SHH to be significantly up-regulated in mNSCs after IFNγ treatment. Interestingly, we could not detect that phenomenon in hNSCs. Another IFNγ-induced phenomenon on a murine background was a strong reduction of the population size of undifferentiated mNSCs. However, we were not able to detect a significant decrease in the population size of hNSCs after IFNγ exposure (Figure 3B). Data were generated with the Wicell H9 line. We also confirmed these findings by using the HUES 6 line (data not shown).

### IFNγ is up-regulated in the cerebrospinal fluid of patients suffering from different diseases

In contrast to the extensively described up-regulation of IFNγ in animal models of human central nervous system diseases, there is only very limited information about amounts of IFNγ in human individuals suffering from neurological diseases. Therefore, we measured the concentration of IFNγ in cerebrospinal fluid (CSF) specimens, taken after lumbar punction from patients suffering from multiple sclerosis, peripheral nervous plexus affections, Alzheimer dementia, viral myelitis, or stroke. We found, that patients suffering from multiple sclerosis during an acute relapse showed a significant increase of IFNγ (three control samples vs. three MS samples). Due to the limitation of patient material, we could only screen single patients with other diseases (one patient per other indicated disease). But also here we found a clear trend toward an up-regulation of IFNγ in the CSF (Figure 3C).

## Discussion

We found here that IFNγ has species-dependent effects on embryonic stem cell-derived neural populations. While this pro-inflammatory key player induces a striking dysregulation in undifferentiated murine neural stem cells (mNSCs) with an unusual coexpression of neuronal and glial markers (Walter et al., 2011, 2012), it has no such effects on human NSCs although both cell populations express appropriate IFNγ receptors and up-regulate most of the classical down-stream signals like i-NOS, IRF-9, c-Myc, MHC 1, and Stat 1, which are known to mediate important IFNγ effects (Schroder et al., 2004).

One possible explanation for this diverse reaction of murine or human NSCs toward IFNγ exposure might be subtle differences in their developmental state or brain region-specific differentiation although both populations consist of proliferating nestin- and Sox2-positive neural precursors. However, the differentiation protocols used in this study were similar for both species leading to a heterogeneous population of neural cells without preference of distinct brain-region specific phenotypes (Illes et al., 2009; Lappalainen et al., 2010; Yla-Outinen et al., 2010). To account for developmental differences, we tested additional IFNγ treatment paradigms for human NSCs including a 7-days treatment or a 5-fold IFNγ concentration but could not detect an up-regulation of neuronal or glial markers or even the appearance of cells that synchronously express these markers (data not shown).

Another explanation for the divergent reactions after IFNγ exposure might be a qualitatively different induction of down-stream signals. While SHH is significantly up-regulated after IFNγ treatment in mNSC populations, it remains unaltered in hNSC populations. Interestingly, recently published results of our group show that the IFNγ-induced dysregulated murine phenotype depends on both, Stat 1 and SHH signaling (Walter et al., 2012). Thus, the lack of an important down-stream signal in the human situation might explain the species-dependent differences. As it is known that human gliomas and tumorigenic NSCs express SHH (Ehtesham et al., 2007) and comprise cells that simultaneously express neuronal and glial markers (Katsetos et al., 2001; Ignatova et al., 2002; Singh et al., 2003; Walton et al., 2009), this morphogene was associated with brain tumor formation and/or growth. Interestingly, in mice a link between IFNγ and SHH signaling was observed as an ectopic expression of IFNγ was shown to induce medulloblastoma formation via SHH overexpression (Lin et al., 2004). Our results in general point to the fact that, possibly fundamental differences in cytokine-induced signaling pathways between human and mouse lead to significant differences in the development of cellular phenotypes. Nevertheless, this type of fundamental differences can also be observed within a single genetic background if, for instance, merely the developmental age of a given cellular population differs. In this regard, several studies investigated the role of IFNγ in human lymphocyte activation and found that, depending on their developmental age (neonatal vs. adult); IFNγ induces divergent down-stream signaling pathways leading to significant differences in their ability to response to pathogens (Wilson et al., 1986; Marodi et al., 1994, 2001; Marodi, 2002). Discussed mechanisms for this divergent response of neonatal or adult lymphocytes to IFNγ are a less effective Stat 1 phosphorylation in neonatal cells or an up-regulation of a new class of cytokine signaling suppressors that can inhibit JAK-Stat pathway (Endo et al., 1997; Starr et al., 1997). In summary, our results admittedly reduce the relevance of an IFNγ-induced dysregulation of undifferentiated NSCs in a human genetic background. However, regarding the above mentioned considerations, it appears to be possible that human NSCs of a different developmental stage in comparison to those we used here can still react to IFNγ exposure with an even misguided initiation of differentiation programs. Thus, our results can rather be interpreted in a way that a given cellular population *in vitro*, even on a human genetic background, might overemphasize results as it not reflects the complexity of an organism. This consideration is substantiated by observations from Johansson et al. (2008). In their study, immortalized hippocampal or striatal human neural stem/progenitor cells from 12-weeks-old fetal brains showed increased neurogenesis and MHC 1 expression after IFNγ exposure during their differentiation phase without growth factors. The divergent results in comparison to our study might simply be explained by the developmental stage of the population during IFNγ exposure as we used proliferating human NSCs under the influence of growth factors. That human fetal cells are principally able to coexpress GFAP and βIII-tubulin under non-inflammatory conditions in the ventricular and subventricular zones as well as under culture conditions was shown by Draberova et al. (2008). Probably this geno- and phenotypic rare case is only possible in a specific time frame of human fetal development and independent from inflammatory stimulation.

To further substantiate the relevance of IFNγ during human CNS diseases, we verified the up-regulation of IFNγ in the CNS. We demonstrate here that patient CSF contains elevated amounts of IFNγ in comparison to control CSF of healthy individuals. Significantly elevated IFNγ levels were found during relapses in multiple sclerosis (*n* = 3) and we also found increased levels in individual patients suffering from peripheral nervous plexus affections, Alzheimer dementia, viral myelitis, or stroke (*n* = 1, respectively). This is in accordance with the fact that human peripheral lymphocytes can secrete up to approximately 200–500 U/ml IFNγ under neuroinflammatory conditions (Hirsch et al., 1985; Chan et al., 1991). As brain ependymal cells lack tight junctions, the CSF compartment in the brain ventricles exchanges neuroactive substances with the interstitial fluid of the brain parenchyma, including neurogenic zones (Alvarez-Buylla and Lim, 2004; Shen et al., 2008; Tavazoie et al., 2008; Ming and Song, 2011; Hartung and Dihne, 2012). This might point to a possible anatomical relationship between IFNγ accumulation within the CSF and NSCs within neurogenic zones, substantiating the relevance of IFNγ effects on NSCs in humans. In summary, our results demonstrate that data collected on a murine genetic background cannot automatically be translated to the human situation and that even on a human background results might differ depending on the developmental stage of the population and their maturation.

## Materials and methods

### Cell culture

The human NSCs used in this study were either derived from the human embryonic stem cell lines HUES 6 (hESC facility; Harvard University; Cambridge, MA, USA) or purchased in a neuroectodermal, predifferentiated form of the human embryonic stem cell line H9 (WiCell Research Institute, Madison, WI, USA/Invitrogen, Karlsruhe, Germany). The detailed neuronal differentiation protocol is described elsewhere (Dhara et al., 2008). Use of hESCs for derivation of neural progenitors was approved by the regulatory authorities at the Robert Koch Institute, Berlin, Germany, and derivation of neural progenitors was performed as follows: the hESCs were cultivated in knockout serum replacement (KSR) media (Cowan et al., 2004) on gelatin coated plates and a feeder layer of γ-irradiated mouse embryonic fibroblasts. Neural differentiation was performed by a protocol previously described by Lappalainen et al. (2010). Briefly, hESCs were dislodged and separated from the feeder cells and afterwards dissected into clusters that contained in between 1000 and 3500 cells. These clusters were placed in neural differentiation/proliferation media that consisted of 1:1 DMEM/F12 and Neurobasal media supplemented with 2 mM GlutaMax, 1XB27, 1XN2, 0.25% BSA, 0.1 mM non-essential amino acids, 25 U/ml penicillin/streptomycin (all products Invitrogen, Karlsruhe, Germany) and 20 ng/ml bFGF (Tebu-bio, Le Perray en Yvelines Cedex, France). Cells were propagated for 8–10 weeks with a medium change every 2–3 days, the formed clusters were dissected once per week. After this time period, the vast majority of cells expressed NSC markers like nestin and Sox 2. Cells were then frozen. For experiments, human NSC were thawn and single cells were seeded on culture surfaces that were coated with poly-L-ornithine (PLO; 0.001%; Sigma-Aldrich, Munich, Germany) and fibronectin (20 μg/ml; BioPur, Bubendorf, Switzerland). In parallel, we used the neuroectodermally pre-differentiated hNSC line (hNSC H9) for comparison. HNSC H9 cells were cultivated and propagated in the same way and in the same media as described above. Human NSCs derived from the cell line H9 were cultivated as free floating clusters and were dissected to single cells for experiments. The attached cells were either treated with human recombinant IFNγ (1000 U/ml; Millipore, Billerica, USA) or kept under control conditions for 3 days. This amount is equal to 100 ng/ml recombinant protein (murine and human). For differentiation experiments, cells had a day of recovery after seeding, then bFGF was withdrawn from the cells and differentiation was performed up to 3 months.

### Immunocytochemistry

For immunocytochemistry, cells were seeded on coated cover slips (VWR International, Darmstadt, Germany). After 3 days under the influence bFGF (20 ng/ml both Tebu-bio) and under different treatments as indicated, the cells were fixed with 4% PFA (Roti-Histofix, Carl Roth, Karlsruhe, Germany) for 15 min at room temperature. Cells were blocked for 30 min at room temperature with 1 fold Roti-Immuno-Block containing 0.25% Triton X-100 for cell wall permeabilization (Carl Roth, Karlsruhe, Germany). Primary antibodies used at 4°C overnight were against βIII-tubulin (Tuj1; 1:500; R&D Systems, Minneapolis, USA), glial fibrillary acid protein (GFAP) (1:500; Dako, Hamburg, Germany), Sox2 (1:50; R&D Systems, Minneapolis, USA), IFNγ-R1 (1:500 Santa Cruz Biotechnology, Heidelberg, Germany), IFNγ-R2 (1:500; Santa Cruz Biotechnology, Heidelberg, Germany) and nestin (1:200; Covance, Munich, Germany). For detection of primary antibodies, fluoresceine-isothiocyanate (FITC; 1:500; Millipore, Billerica, USA) or indocarbocyanine (Cy3; 1:800; or Cy5; 1:200; Millipore, Billerica, USA) coupled secondary antibodies were used. The first and secondary antibodies were diluted in 1-fold Roti-Immuno-Block without Triton X-100 (Carl Roth, Karlsruhe, Germany). For visualization of cell nuclei cells were co-stained with DAPI (Invitrogen, Karlsruhe, Germany). For negative controls, primary antibodies were omitted in each experiment.

### MTT-assay

To analyze the population size of hNSCs, the optical density, indicative of conversion of 3-(4, 5-dimethylthiazol-2-yl)-2, 5-diphenyltetrazolium bromide (MTT; Sigma-Aldrich) into formazan crystals which takes place in live cells only, was determined after IFNγ treatment at indicated concentrations. An OD value of 0.5 represents approximately 50,000 and an OD value of 1.0 represents approximately 100,000 live NSCs. The population size was measured after the indicated time intervals. Therefore, MTT (Invitrogen, Karlsruhe, Germany) was added to the cell culture media at a final concentration of 0.5 mg/ml after 5 h of incubation media was skipped and DMSO (Invitrogen, Karlsruhe, Germany) was added for the solubilization of formed crystals. We seeded the same amount of cells at day 0 and performed an MTT-Assay on day 3, 5, and 7. At every time point control cells and cells treated with human recombinant IFNγ were tested. We used the same dose of human recombinant IFNγ, which was used in the murine study (1000 U/ml).

### Real-time quantitative PCR

RNeasy Kit (Qiagen, Hilden, Germany) was used for RNA isolation of cultured NSCs. Then a reverse transcription into cDNA (ABI, Darmstadt, Germany) was performed. Real-time quantitative PCR was carried out by the usage of the 7500 fast or 7500 real-time quantitative PCR cycler (ABI, Darmstadt, Germany). SYBR green master mix (Qiagen, Hilden, Germany) or equivalent chemistry from another supplier (Quantace, London, UK) was used. The specific primers for genes of interest or the housekeeping gene were either purchased (QuantiTect primer assays, Qiagen) or self-designed (BioTEZ, Berlin, Germany). The genes of interest (target gene) in IFNγ-treated groups or control groups (PBS-treated) were analyzed in at least 3 independent cultures in triplicate each. Every experiment in IFNγ-treated or control (PBS-treated) groups provided delta CT values (ΔCT: gene of interest minus reference gene).

### Statistical analyses

Experiments were repeated with independent cultures at least three times in triplicate each. The resulting data sets were statistically analyzed und illustrated using the GraphPad Prism 4 (GraphPad Software Inc., San Diego, CA, USA, 2003) software. For approval of statistical significance between groups, a two-tailed unpaired *t*-test was performed. *P*-values <0.05 were considered to indicate significant differences.

### CSF collection and IFNγ ELISA

Lumbar CSF was obtained from patients admitted to our department for diagnostic purposes. Patients suffered from multiple sclerosis (three samples), peripheral nervous plexus affections (one samples), Alzheimer's disease (one sample), viral myelitis (one sample), or stroke (one sample), were compared to control CSF (three samples). All CSF specimens were immediately centrifuged, aliquoted and stored at –35°C. Ethics approval for the use of human CSF was obtained from the institutional ethics committee. For IFNγ detection in the CSF samples an IFNγ ELISA by R&D systems (R&D systems, Wiesbaden-Nordenstadt, Germany) was used. The experimental protocol followed the manufacturer's instructions. For statistical testing 3 control patients were compared with 3 MS patients with an unpaired two-tailed *t*-test.

### Conflict of interest statement

The authors declare that the research was conducted in the absence of any commercial or financial relationships that could be construed as a potential conflict of interest.
